# PI3K/Akt signaling pathway mediates the effect of low-dose boron on barrier function, proliferation and apoptosis in rat intestinal epithelial cells

**DOI:** 10.1038/s41598-023-50800-2

**Published:** 2024-01-03

**Authors:** Shuqin Chen, Jialiang Huang, Ting Liu, Feng Zhang, Chunfang Zhao, Erhui Jin, Shenghe Li

**Affiliations:** 1 College of Animal Science, Anhui Science and Technology University, No. 9, Donghua Road, Fengyang County, Chuzhou City, Anhui Province China; 2 Anhui Province Key Laboratory of Animal Nutritional Regulation and Health, No. 9, Donghua Road, Fengyang County, Chuzhou City, Anhui Province China

**Keywords:** Physiology, Environmental sciences, Gastroenterology, Health care

## Abstract

Boron is an essential trace element with roles in growth, development, and physiological functions; however, its mechanism of action is still unclear. In this study, the regulatory roles of the PI3K/Akt signaling pathway on boron-induced changes in barrier function, proliferation, and apoptosis in rat intestinal epithelial cells were evaluated. Occludin levels, the proportion of cells in the G2/M phase, cell proliferation rate, and mRNA and protein expression levels of PCNA were higher, while the proportions of cells in the G0/G1 and S phases, apoptosis rate, and caspase-3 mRNA and protein expression levels were lower in cells treated with 0.8 mmol/L boron than in control IEC-6 cells (*P* < 0.01 or *P* < 0.05). However, 40 mmol/L boron decreased ZO-1 and Occludin levels, the proportion of cells in the G2/M phase, cell proliferation rate, and mRNA and protein levels of PCNA and increased the apoptosis rate and caspase-3 mRNA expression (*P* < 0.01 or* P* < 0.05). After specifically blocking PI3K and Akt signals (using LY294002 and MK-2206 2HCL), 0.8 mmol/L boron had no effects on Occludin, PCNA level, apoptosis rates, and caspase-3 levels (*P* < 0.05); however, the proliferation rate and PCNA levels decreased significantly *(P* < 0.01 or *P* < 0.05). The addition of 40 mmol/L boron did not affect ZO-1 and Occludin levels and did not affect the apoptosis rate or PCNA and caspase-3 levels. These results suggested that the PI3K/Akt signaling pathway mediates the effects of low-dose boron on IEC-6 cells.

## Introduction

Boron is a common mineral element in the environment, with about 10 g per ton of rock in the earth's crust and inorganic or organic forms in most soils^1^. Boron and its compounds are widely used in materials science, engineering, agriculture, and medicine. In daily life, boron compounds are raw materials for detergents, preservatives, insecticides, and accelerants^2,3^. Recent studies have shown that boron is an indispensable trace element for animals. A lack of boron can cause calcium and phosphorus losses, leading to osteoporosis and immune dysfunction^4^. Dietary supplementation with appropriate doses of boron can improve a variety of physiological functions in animals^5^. Adding a proper dose of boron to drinking water can enhance immune function as well as absorption and metabolism in the intestine of rats^6^, promote the development of the bone and thymus gland of ostrich and chicken, improve liver function^7^. And the addtion of a proper dose of boron in daily diet can improve calcium and phosphorus metabolism in dairy cows, and relieve postpartum paralysis in dairy cows^8^. However, high doses of boron or exposure to high levels of boron for long periods of time cause physiological injury. Jin et al.^9^ found that adding 320 or 640 mg/L boron to drinking water inhibits thymus development in rats, significantly reduces the number of lymphocytes, significantly decreases immune and antioxidant indexes, and significantly increases thymic cell apoptosis. The intestinal epithelial barrier is a one-cell-thick internal lining of the gut that contains different types of epithelial cells. Studies have demonstrated that a variety of intestinal diseases are associated with intestinal mucosal barrier damage ^10–12^.Therefore, therapeutic and nutritive intervention for the regulation of barrier integrity is an emerging topic and more investigations are essential to understand the role of intestinal barrier integrity in various diseases^13^.

The intestinal tract an important organ for digestion, absorption, and general health. We have previously found that drinking water supplemented with 40 and 80 mg/L boron can significantly increase the villus height and crypt depth of the duodenum, the number of intestinal goblet cells, the intraepithelial lymphocytes, and the related immunoglobulin contents in rats. Besides, the intestinal growth and development and barrier function are also enhanced in rats. However, supplementation with 320 mg/L or 640 mg/L boron can damage the intestinal tissue structure and inhibit growth and development^14^. These results strongly suggest that boron at various doses can significantly affect intestinal barrier function in animals.

Phosphatidylinositol kinase/Protein kinase B (PI3K/Akt) is an important intracellular signaling pathway involved in the regulation of cell proliferation, apoptosis, differentiation, motility, and other processes^15^. Yan et al. found that PI3K/Akt is an important pathway in intestinal mucosal injury repair. It can regulate intestinal tight junction protein synthesis and intestinal barrier integrity, promote the proliferation of pig intestinal epithelial cells (IPEC-J2), and improve intestinal barrier function^16^. However, it is not clear whether the effects of boron on intestinal barrier function are mediated by the PI3K/Akt signaling pathway. Therefore, in this study, specific blockers were used to inhibit the PI3K and Akt signaling pathways to evaluate the effects of boron on barrier function, proliferation, and apoptosis in small intestinal epithelial cells in rats, providing insight into the mechanism by which boron contributes to physiological functions in animals.

## Materials and methods

### Cells and reagents

The following cells and reagents were used: rat small intestinal epithelial cell line IEC-6 (National Laboratory Cell Resource Sharing Platform, China), RIPM-1640 (HyClone, Logan, UT, USA), fetal bovine serum (FBS) (GIBCO, Victoria, Australia), Penicillin–streptomycin (HyClone, USA), Boric acid (H_3_BO_3_) (Cat: 20,120,524, Sinopharm Group, Beijing, China); Cell Counting Kit-8 (Abbkine, Wuhan, China), FITC Apoptosis Detection Kit (BD Pharmingen, San Diego, CA, USA), PI/RNase Tillering Buffer Kit (BD Pharmingen, USA), PI3K Specific Blocker LY294002 (Selleck Chemicals, Houston, TX, USA); Akt Specific Blocker MK-2206 2HCL (Selleck Chemicals, USA), zonula occludens-1 (ZO-1) and Occludin enzyme-linked immuno sorbent assay (ELISA) kits (Dakome Technology, Shanghai, China), Cell Total RNA Extraction Kit (DP430, Tiangen, Beijing, China), Reverse Transcription Kit (Thermo Scientific, Waltham, MA, USA),; TB Green Premiere Ex Taq (Takara Bio, Kusatsu, Japan); Rabbit Anti- proliferating cell nuclear antigen (PCNA) Polyclonal Antibody, Rabbit Anti-Caspase-3 Polyclonal Antibody, Rabbit Anti-rat β-actin Antibody (Abcam, Cambridge, MA, USA), and Bicinchoninic acid Protein Determination Kit (Solarbio, Beijing, China).

### Culture and treatment of rat intestinal epithelial cells (IEC-6)

IEC-6 cells were cultured in medium containing 89% RPMI 1640, 10% FBS, and 1% penicillin + streptomycin. According to experimental requirements, IEC-6 cells were divided into nine groups (three replicates per group) as follows: 0 mmol/L boron (CK), 0.8 mmol/L boron (LD), 40 mmol/L boron (HD), 10 µmmol/L LY294002, 0.8 mmol/L boron + LY294002 (LDLY), 40 mmol/L boron + LY294002 (HDLY), 10 µmmol/L MK-2206 2HCL, 0.8 mmol/L boron + MK-2206 2HCL (LDMK), and 40 mmol/L boron + MK-2206 2HCL (HDMK). After cells were resuscitated, they were cultured to the exponential growth stage and collected. The cell concentration was adjusted to 10^6^ cells/well and cultured at 37 °C with 5% CO_2_ for 48 h. The cells were treated with 10 µmol/L LY294002 or MK-2206 2HCL for 3 h and then cultured with different doses of boron for 24 h. All experimental procedures were conducted in strict accordance with the provincial “Guide for the Care and Use of Laboratory Animals”and the “National Guide for the Care and Use of Laboratory Animals.”

### ELISA

The cell supernatant was collected by centrifugation, and the contents of ZO-1 and Occludin were detected using ELISA kits, following the manufacturer’s procedure. Absorbance was measured by an elisa plate reader (Multiskan GO, Thermo Scientific, USA), and the correlation coefficients of the standard curves (i.e., linearity) for ZO-1 and Occludin were 0.9962, 0.9948, and 0.9937, respectively.

### Flow cytometry

Cells were collected by centrifugation, rinsed with 1% phosphate-buffered saline (PBS), and stained with 5 µL of Annexin V-FITC and 5 µL of propidium iodide (PI) for 15 min. Then, the rate of apoptosis was measured by flow cytometry (BD FACSCalibur, BD Pharmingen) using a 300-mesh filter.

The cells were collected and soaked in 70% ethanol at 4 °C for 30 min. The cells were washed with 1% PBS, soaked with 50 mg/mL PI at 25 °C for 30 min, and then cultured with 100 mg/mL RNase A (37 °C, 2% CO_2_) for 1 h. Cell cycle changes were detected by flow cytometry.

### Real-time fluorescence quantitative PCR (q-PCR)

Cells were collected by centrifugation and total RNA was extracted using the Cell Total RNA Extraction Kit. The RNA concentration was detected using the NanoDrop One spectrophotometer (Thermo Scientific), and the integrity of total RNA was detected by agarose gel electrophoresis. cDNA was synthesized from RNA using the Thermo Scientific Reverse Transcription Kit following the manufacturer’s instructions. The amplification system was prepared according to the operating instructions of the real-time quantitative PCR kit, and qPCR amplification was performed on the real-time quantitative PCR instrument (Roche LightCycler® 480II, Kanton Basel-Stadt, Switzerland). Real-time PCR amplification parameters were set as follows: 95 °C for 5 min, 95 °C for 15 s, 60 °C for 15 s, and 72 °C for 15 s (45 cycles) using specific primers (Supplementary Table 1). Each sample was measured three times. Glyceraldehyde 3-phosphate dehydrogenase (*GAPDH*) was used as a reference gene to ensure the accuracy of q-PCR data. The relative expression levels of target genes were calculated by the 2^-△△Ct^ method.

### Western blotting

Cells were collected by centrifugation, washed with pre-cooled 1% Phosphate Buffered Saline (PBS) twice, and suspended with radio immunoprecipitation assay Lysis Buffer. Centrifugation was performed at 4 °C and 12,000 rpm for 10 min. The supernatant was collected, and the total protein concentration was determined using a Solarbio Bicinchoninic acid protein determination kit. Proteins were isolated by SDS-PAGE and transferred to a PVDF membrane by the wet transfer method. Then, the PVDF membrane was placed in 5% skim milk powder and sealed at 37 °C for 2 h. The primary antibody and HRP-labeled secondary antibody were added and incubated at room temperature for 1 h. Western blot exposure was performed (Alpha Innotech, San Leandro, CA, USA). The bands were obtained by Quantity One to evaluate optical density, and the relative protein expression was calculated by the formula: Ratio = integrated optical density of target protein/integrated optical density of internal reference protein (i.e., β-actin).

### Statistical analysis

Statistical analyses were performed using SPSS 18.0 (SPSS Inc., Chicago, IL, USA) and results were expressed as mean ± standard deviation (M ± SD). The Levene test and Kolmogorov − Smirnov test were used to determine the homogeneity and normality of the data. The LSD test was used to analyze differences in mean values between groups. *P* < 0.05 indicated significant difference, and *P* < 0.01 indicated an extremely significant difference. Comparisons were performed between the following treatment groups: LD or HD to CK, LY or MK to CK, LDLY or HDLY to LY, LDMK or HDMK to MK.

## Results

### Effects of boron on ZO-1 and Occludin secretion in IEC-6 cells after PI3K and Akt blockade

**Figure 1:**
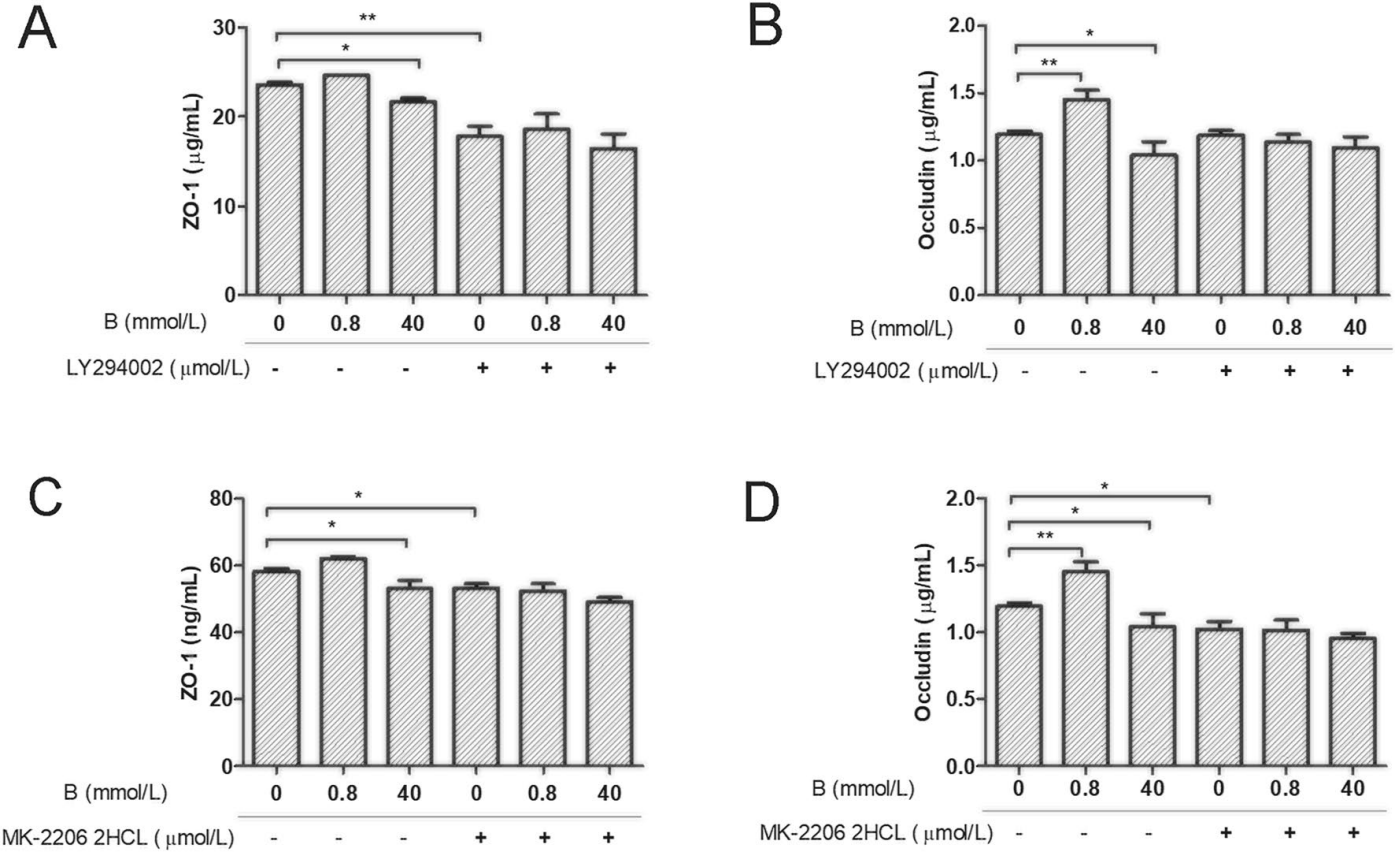
The effects of boron on tight junction protein secretion in rat IEC-6 cells before and after PI3K/Akt inhibition. ELISA was used to analyze the effect of boron on secretion of ZO-1 and Occludin in IEC-6 cells before and after PI3K and Akt were inhibited by LY294002 and MK2206 2HCL, respectively. (**A**,**B**): IEC-6 cell culture medium was supplemented with 0, 0.8, 40 mmol/L boron and 10 μmol/L LY249002, respectively; (**C**,**D**): IEC-6 cell culture medium was supplemented with 0, 0.8, 40 mmol/L boron and MK2206 2HCL of 10 μmol/L, respectively; Values are presented as the mean ± SD; Mean values with asterisk (*) differed significantly (*p* < 0.05), mean values with two asterisks (**) indicate extremely significant differences (*p* < 0.01).

As shown in Fig. 1, levels of Occludin (Fig. 1B) in IEC-6 cells in the LD group were significantly higher than those in the CK group (*P* < 0.01). The ZO-1 (Fig. 1A) and Occludin contents in IEC-6 cells were significantly decreased in the HD group than those in the CK group (*P* < 0.05 or *P* < 0.01).

After adding 10 μmol/L LY294002, the ZO-1 (Fig. 1A) content in IEC-6 cells in the LY group was significantly lower than that in the CK group (*P* < 0.01), and there were no significant differences in ZO-1 (Fig. 1A) and Occludin (Fig. 1B) levels in IEC-6 cells between the LDLY and HDLY groups and the LY group (*P* > 0.05). After the addition of 10 μmol/L MK-2206 2HCL, the contents of ZO-1 (*P* < 0.05) (Fig. 1C), and Occludin (*P* < 0.05) (Fig. 1D) in IEC-6 cells in the MK group were significantly lower than those in the CK group. However, there were no significant differences in ZO-1 and Occludin in IEC-6 cells among the LDMK, HDMK, and MK groups (*P* > 0.05) (Fig. 1C,D).

The results showed that 0.8 mmol/L boron did not significantly increase the contents of Occludin in IEC-6 cells after the specific inhibition of PI3K and Akt signals. The addition of 40 mmol/L boron did not significantly reduce the contents of ZO-1 and Occludin in IEC-6 cells. These findings suggested that PI3K and Akt mediated the effect of 0.8 mmol/L and 40 mmol/L boron on the expression of Occludin in IEC-6 cells.

### Effect of boron on the cell cycle progression after PI3K or Akt blockade

**Figure 2:**
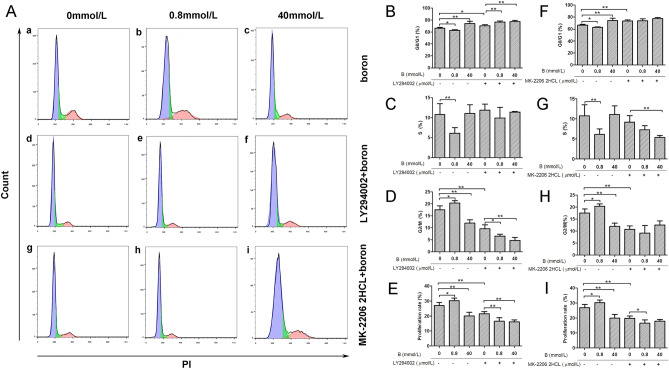
The effects of boron on cell cycle and proliferation rate before and after PI3K/Akt inhibition. Flow cytometry was used to analyze the effects of boron on the phases of G0/G1, S, G2/M and proliferation rate of IEC-6 cells before and after PI3K and Akt were inhibited by LY294002 and MK-2206 2HCL, respectively. (**A**) (Flow chart) (a–c) IEC-6 cultured without LY294002 or MK-2206 2HCL; (d–f) IEC-6 cultured with 10 μmol/L LY294002; (g–i) IEC-6 cultured with 10 μmol/L MK-2206 2HCL; 0 mmol/L boron (a, d, g), 0.8 mmol/ L boron (b, e, h) and 40 mmol/L boron (c, f, i); (**B**)The changes in the ratio of G0 phase cell number to G1 phase cell number before and after the addition of LY249002; (**C**)The changes of cell number in S phase before and after the addition of LY249002; (**D**)The changes in the ratio of G2 phase cell number to M phase cell number before and after the addition of LY249002; (**E**) The IEC-6 proliferation rate before and after LY294002 addition; (**F**)The changes in the ratio of G0 phase cell number to G1 phase cell number before and after the addition of MK-2206 2HCL; (**G**)The changes of cell number in S phase before and after the addition of MK-2206 2HCL; (**H**)The changes in the ratio of G2 phase cell number to M phase cell number before and after the addition of MK-2206 2HCL; (**I**) The IEC-6 proliferation rate before and after MK2206 2HCL addition.Values are presented as the mean ± SD; Mean values with asterisk (*) differed significantly (*p* < 0.05), mean values with two asterisks (**) indicate extremely significant differences (*p* < 0.01).

As shown in Fig. 2, compared with those in the CK group, the proportions of G0/G1 (Fig. 2Aa–c, B) and S phase cells (Fig. 2Aa–c, C) in the LD group were significantly lower (*P* < 0.05 or *P* < 0.01), while the proportion of G2/M phase cells (Fig. 2Aa–c, D) and rate of cell proliferation were significantly higher (*P* < 0.05). The proportion of G0/G1 phase cells (Fig. 2Aa–c, B) in the HD group was significantly increased (*P* < 0.01), the proportion of G2/M phase cells (Fig. 2Aa–c, D) and the rate of proliferation (Fig. 2E)were significantly decreased (*P* < 0.01), and the proportion of S phase cells (Fig. 2Aa–c, D) had no significant change (*P* > 0.05).

After the addition of 10 µmol/L LY294002, the proportion of G0/G1 phase cells (Fig. 2Ad–f, B) in the LY group was significantly higher (*P* < 0.05) and the proportion of G2/M phase cells (Fig. 2Ad–f, D) and cell proliferation rate (Fig. 2E) were significantly lower (*P* < 0.01) than those in the CK group. The percentages of G1/G0 cells (Fig. 2Ad–f, B) were significantly higher (*P* < 0.01) and the percentage of G2/M (Fig. 2Ad–f, D) cells and cell proliferation rate (Fig. 2E) were significantly lower (*P* < 0.01 or *P* < 0.05) in the LDLY and HDLY groups than in the LY group. The percentage of cells in S phase (Fig. 2Ad–f, C) was not significantly decreased in the LDLY group and the HDLY group than the CK group (*P* > 0.05). After the addition of 10 µmol/L MK-2206 2HCL, the proportion of G0/G1 phase cells (Fig. 2Ag–i, F) was significantly higher (*P* < 0.01) and the proportion of G2/M phase cells (Fig. 2Ag–i, H) and cell proliferation rate (Fig. 2I) were significantly lower (*P* < 0.01) in the MK group than in the CK group. Compared with those in the MK group, the proliferation rate (Fig. 2I) of IEC-6 cells in the LDMK group was significantly lower (*P* < 0.05); the percentage of cells in S phase (Fig. 2Ag–i, G) was also significantly decreased in the HDMK group (*P* < 0.01), while there were no significant changes in GO/G1 (Fig. 2Ag–i, F) and G2/M phase cells (Fig. 2Ag–i, H) (*P* > 0.05).

The specific inhibition of PI3K or Akt signaling significantly increased the proportion of G0/G1 cells and significantly decreased the proportion of G2/M cells and rate of cell proliferation. After the specific inhibition of PI3K signaling, 0.8 mmol/L boron supplementation did not significantly reduce the proportion of S-phase cells but significantly increased the proportion of G2/M cells and cell proliferation rate. The addition of 40 mmol/L boron significantly increased the proportion of G0/G1 cells and significantly decreased the proportion of G2/M cells and cell proliferation rate. After the specific inhibition of Akt signaling, 0.8 mmol/L boron supplementation did not significantly reduce the proportion of G0/G1 and S phase cells and did not increase the proportion of G2/M cells and cell proliferation rate. The addition of 40 mmol/L boron did not increase the proportion of G0/G1 cells or decrease the proportion of G2/M cells and cell proliferation rate; however, it significantly decreased the proportion of S phase cells. These results suggested that Akt signaling mediated the effects of 0.8 and 40 mmol/L boron on cell cycle progression in IEC-6.

### Effect of boron on IEC-6 cell apoptosis after PI3K or Akt blockade

**Figure 3:**
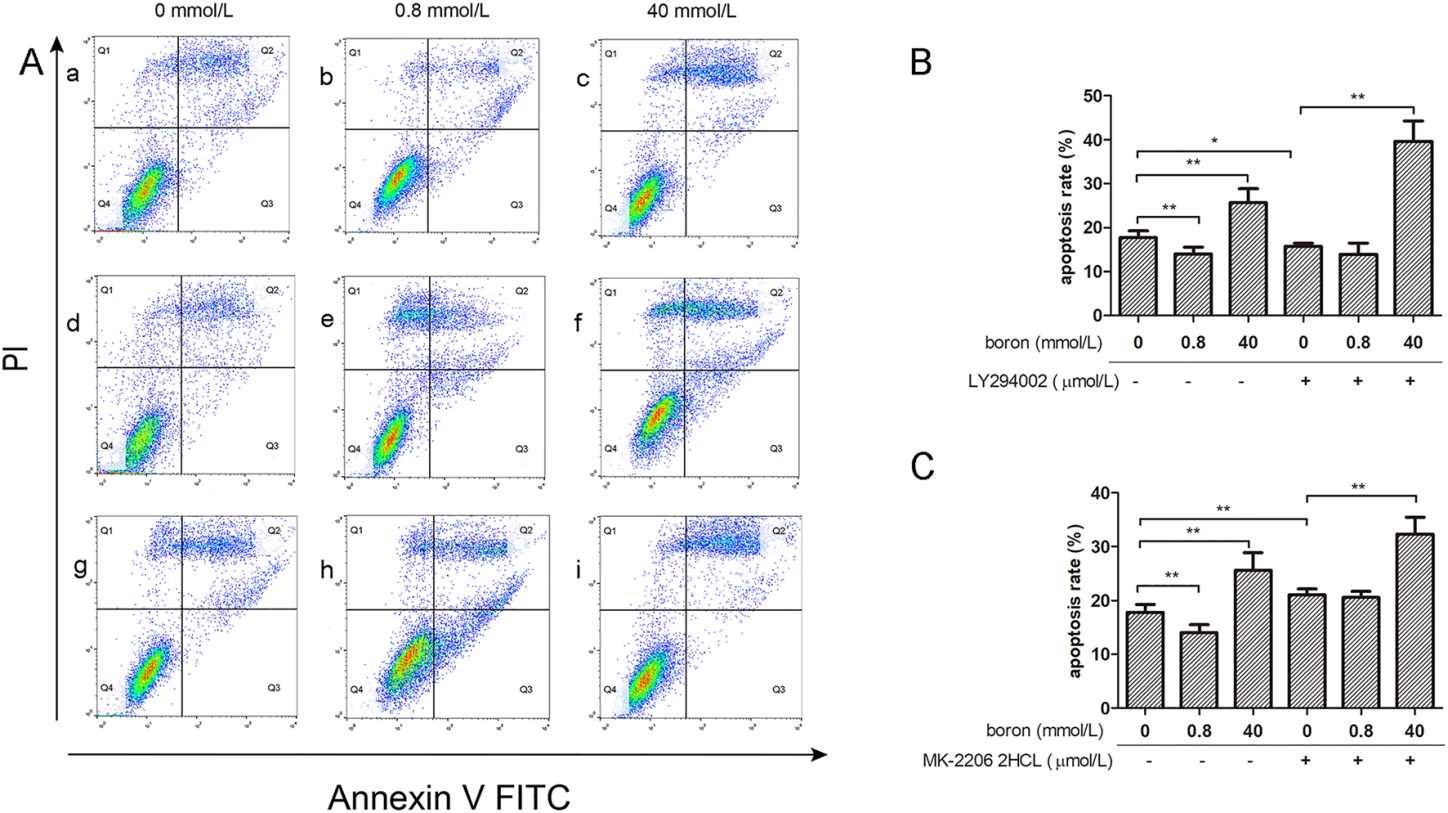
The effects of boron on apoptosis rate before and after PI3K/Akt inhibition. Flow cytometry and CCK-8 were used to analyze the effect of boron on the apoptosis rate of IEC-6 cells before and after PI3K and Akt were inhibited by LY294002 and MK-2206 2HCL, respectively. (**A**) Flow chart for IEC-6 apoptosis detection before and after MK-2206 2HCL addition. (a–c) IEC-6 cultured without LY294002 or MK-2206 2HCL; (d–f) IEC-6 cultured with 10 μmol/L LY294002; (g–i) IEC-6 cultured with 10 μmol/L MK-2206 2HCL; 0 mmol/L boron (a, d, g), 0.8 mmol/ L boron (b, e, h) and 40 mmol/L boron (c, f, i) (**B**) The apoptosis rate of IEC-6 cells before and after LY294002 addition; (**C**) The apoptosis rate of IEC-6 cells before and after MK-2206 2HCL addition; Values are presented as the mean ± SD; Mean values with asterisk (*) differed significantly (*p* < 0.05), mean values with two asterisks (**) indicate extremely significant differences (*p* < 0.01).

As shown in Fig. 3, compared with estimates in the CK group, the number of living cells in the LD group (Fig. 3Aa, Ab) increased, the number of apoptotic cells (Fig. 3Aa–c) decreased, and the rate of apoptosis (Fig. 3B) decreased extremely significantly (*P* < 0.01). The number of living cells decreased and apoptotic cells increased in the HD group (Fig. 3Aa, 3Ac) and the apoptotic rate (Fig. 3B) increased significantly than those in the CK group (*P* < 0.01).

After the addition of 10 µmol/L LY294002, the rate of apoptosis in the LY group (Fig. 3B) was significantly lower than that in the CK group (*P* < 0.05). Compared with that in the LY group, the rate of apoptosis in the LDLY group (Fig. 3B) did not differ significantly (*P* > 0.05), while that of the HDLY group (Fig. 3B) increased significantly (*P* < 0.01). After the addition of 10 µmol/L MK-2206 2HCL, the rate of apoptosis in the MK group (Fig. 3C) was significantly higher than that in the CK group (*P* < 0.01). Compared with that in the MK group, the rate of apoptosis in the LDMK group (Fig. 3C) did not differ significantly (*P* > 0.05); however, the rate of apoptosis in the HDMK group (Fig. 3C) was significantly higher than that in the MK group (*P* < 0.01).

The results showed that 0.8 mmol/L boron supplementation did not significantly reduce the rate of cell apoptosis after the specific inhibition of PI3K or Akt signaling; however, 40 mmol/L boron significantly increased the rate of apoptosis. These results suggest that the PI3K/Akt signaling pathway mediates the effect of 0.8 mmol/L boron on IEC-6 cell apoptosis; however, it does not mediate the effect of 40 mmol/L boron on IEC-6 cell apoptosis.

### Effects of boron on *PCNA* and Caspase-3 mRNA expression in IEC-6 cells after PI3K or Akt blockade

**Figure 4:**
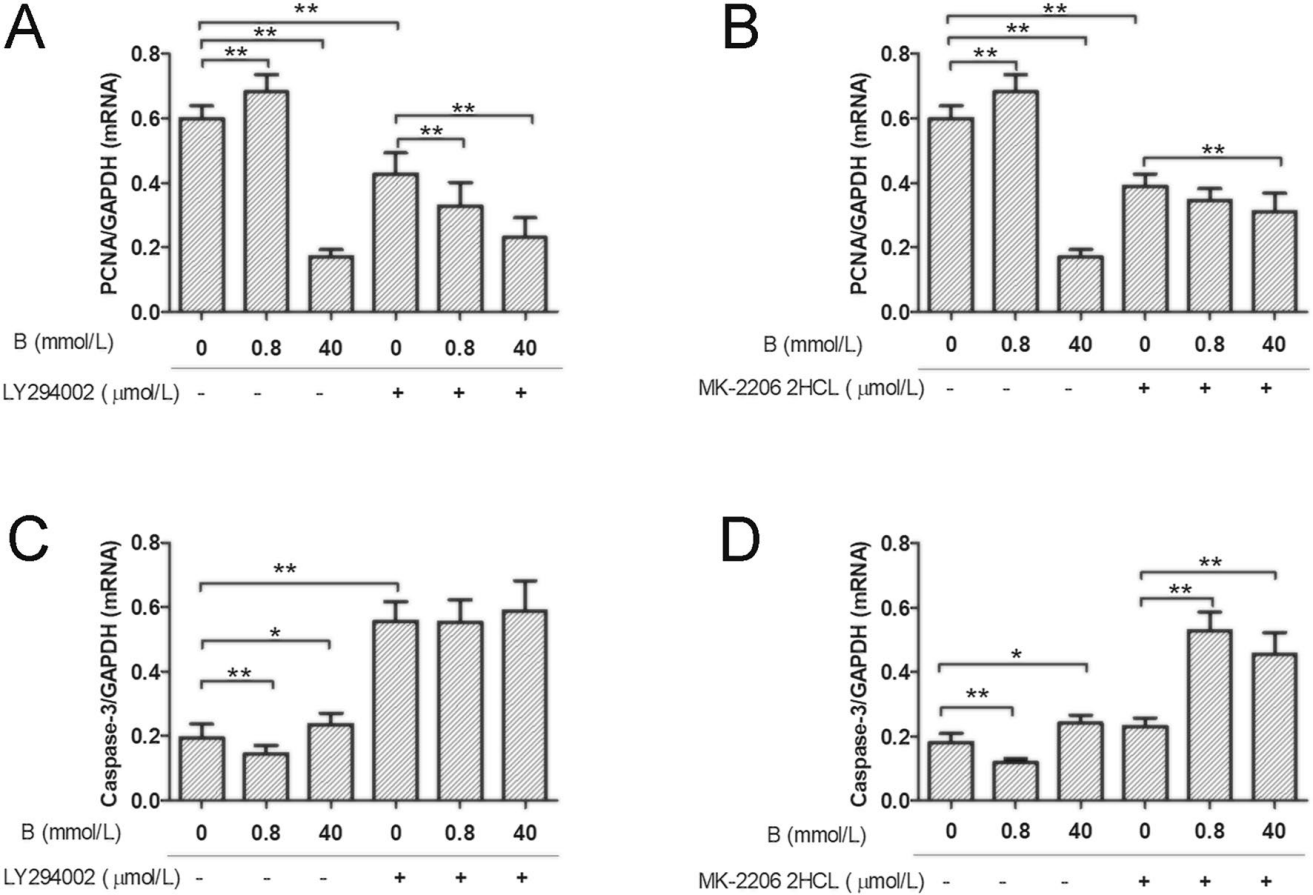
The effects of boron on mRNA expression of proliferation and apoptosis genes before and after PI3K/Akt inhibition. Q-PCR was used to analyze the effects of boron on the expression of *PCNA* and Caspase-3 mRNA in IEC-6 cells before and after PI3K and Akt were inhibited by LY294002 and MK-2206 2HCL, respectively. (**A**) *PCNA* mRNA expression (addition of 10 μmol/L LY294002); (**B**): Caspasse-3 mRNA expression (addition of 10 μmol/L MK2206 2HCL); (**C**) *PCNA* mRNA expression (addition of 10 μmol/L LY294002); (**D**) Caspasse-3 mRNA expression (addition of 10 μmol/L MK2206 2HCL); Values are presented as the mean ± SD; Mean values with asterisk (*) differed significantly (*p* < 0.05), mean values with two asterisks (**) indicate extremely significant differences (*p* < 0.01).

As shown in Fig. 4, *PCNA* mRNA expression levels (Fig. 4A) in IEC-6 cells were significantly higher (*P* < 0.01) and Caspase-3 mRNA levels (Fig. 4C) were significantly lower (*P* < 0.01) in the LD group than in the CK group. The expression of *PCNA* mRNA (Fig. 4A) in IEC-6 cells in the HD group was significantly decreased (*P* < 0.01) and the expression of Caspase-3 mRNA (Fig. 4C) was significantly increased (*P* < 0.05).

After the addition of 10 µmol/L LY294002, *PCNA* mRNA expression levels (Fig. 4A) in IEC-6 cells were significantly lower (*P* < 0.01) and Caspase-3 mRNA levels (Fig. 4C) were significantly higher (*P* < 0.01) in the LY group than in the CK group. Compared with levels in the LY group, *PCNA* mRNA expression (Fig. 4A) of IEC-6 cells in the LDLY group was significantly lower (*P* < 0.01), while caspase-3 mRNA expression (Fig. 4C) did not differ (*P* > 0.05). *PCNA* mRNA levels (Fig. 4A) in IEC-6 cells in the HDLY group were significantly decreased (*P* < 0.01), while caspase-3 mRNA expression levels (Fig. 4C) did not differ significantly (*P* > 0.05). After 10 µmol/L MK-2206 2HCL was added, *PCNA* mRNA levels (Fig. 4B) in IEC-6 cells in the MK group were significantly lower than those in the CK group (*P* < 0.01). Compared with levels in the MK group, the expression of *PCNA* mRNA (Fig. 4B) in the LDMK group did not differ significantly (*P* > 0.05), while the expression of Caspase-3 mRNA (Fig. 4D) was significantly higher (*P* < 0.01). In the HDMK group, *PCNA* mRNA expression levels (Fig. 4B) in IEC-6 cells decreased significantly (*P* < 0.01), while Caspase-3 mRNA expression (Fig. 4D) increased significantly (*P* < 0.01).

*PCNA* mRNA expression decreased significantly and Caspase-3 mRNA expression increased significantly in IEC-6 cells after the specific inhibition of PI3K signaling. Supplementation with 0.8 mmol/L boron did not significantly decrease the expression of Caspase-3 mRNA in IEC-6 cells but significantly decreased the expression of *PCNA* mRNA. The addition of 40 mmol/L boron did not significantly decrease Caspase-3 mRNA expression but significantly decreased *PCNA* mRNA expression. After the specific inhibition of Akt signaling, 0.8 mmol/L boron supplementation did not significantly increase the expression of *PCNA* mRNA in IEC-6 cells but significantly increased Caspase-3 mRNA expression. The addition of 40 mmol/L boron significantly decreased *PCNA* mRNA expression and increased Caspase-3 mRNA expression. These results suggested that the effects of 0.8 mmol/L boron on the mRNA expression of *PCNA* and Caspase-3 in IEC-6 cells were mainly mediated by PI3K and Akt signaling.

### Effects of boron on PCNA and caspase-3 protein levels in IEC-6 cells after PI3K or Akt blockade

**Figure 5:**
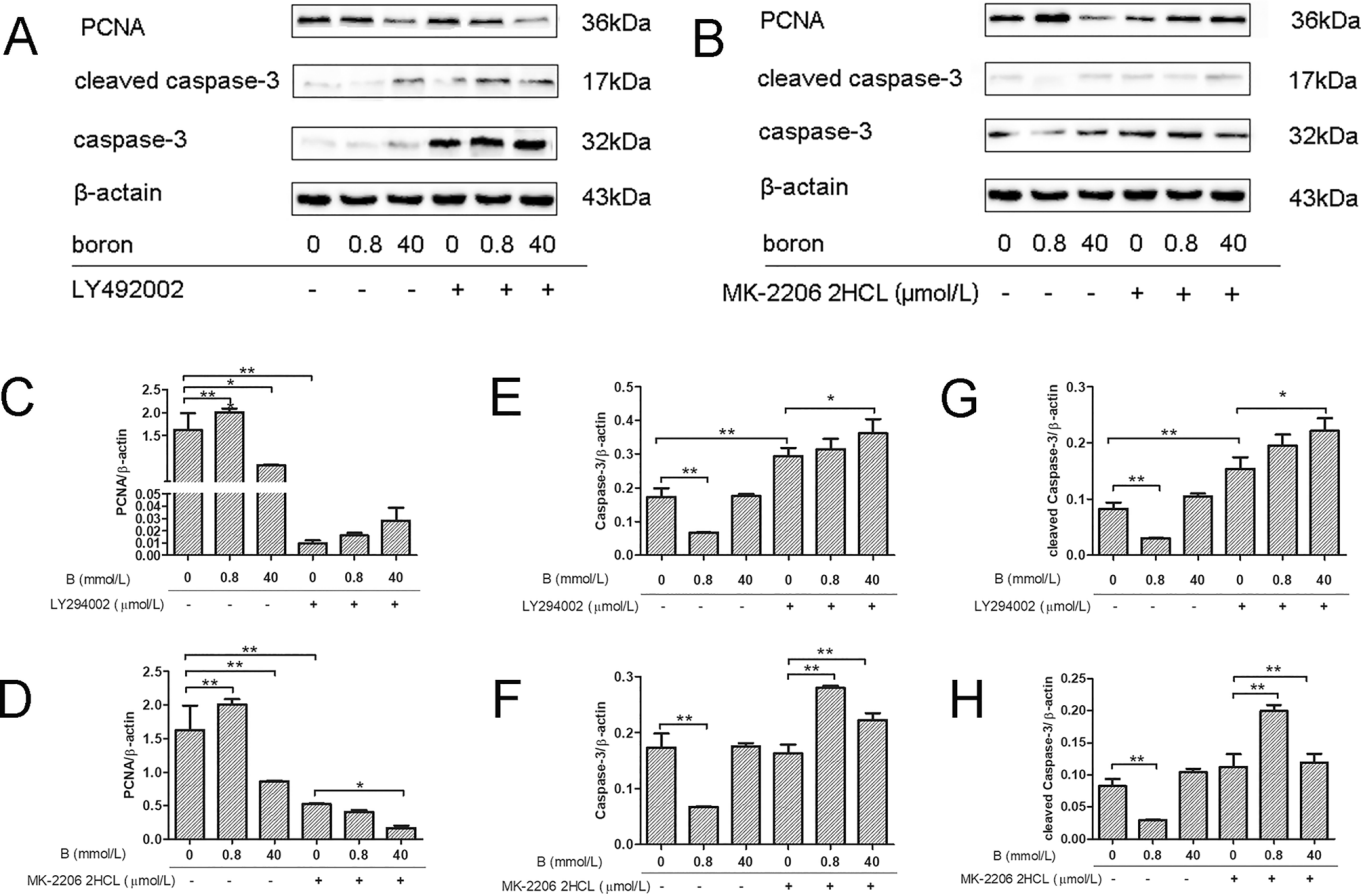
Effects of boron on protein expression of cell proliferation and apoptosis genes before and after PI3K/Akt inhibition. Western-blot was used to analyze the effects of boron on the expression levels of PCNA and Caspase-3 proteins in IEC 6 cells before and after PI3K and Akt were inhibited by LY294002 and MK-2206 2HCL, respectively. (**A**) The western-blot test results for the expressions of Caspase-3 and PCNA proteins in IEC-6 before and after addition of LY294002; (**B**) The western-blot test results for the expressions of PCNA proteins in IEC-6 before and after addition of MK-2206 2HCL.; (**C**) PCNA protein expression (addition of 10 μmol/L LY294002); (**D**) PCNA protein expression (addition of 10 μmol/L MK-2206 2HCL); (**E**) Caspase-3 protein expression (addition of 10 μmol/L LY294002); (**F**) Caspase-3 protein expression (addition of 10 μmol/L MK-2206 2HCL); (**G**) Cleaved Caspase-3 protein expression (addition of 10 μmol/L LY294002); (**H**) Cleaved Caspase-3 protein expression (addition of 10 μmol/L MK-2206 2HCL); Values are presented as the mean ± SD; Mean values with asterisk (*) differed significantly (*p* < 0.05), mean values with two asterisks (**) indicate extremely significant differences (*p* < 0.01).

As shown in Fig. 5, compared with levels in the CK group, PCNA protein expression (Fig. 5A,C) in IEC-6 cells in the LD group was significantly higher (*P* < 0.01), caspase-3 expression (Fig. 5A,E) in the LD group was significantly lower (*P* < 0.01), and PCNA protein expression (Fig. 5A,C) in the HD group was significantly lower (*P* < 0.01). There was no significant difference in caspase-3 protein (Fig. 5A,E) expression among groups (*P* > 0.05).

After the addition of 10 µmol/L LY294002, compared with levels in the CK group, PCNA protein expression (Fig. 5A,C) in IEC-6 cells in the LY group was significantly lower (*P* < 0.01), cleaved Caspase-3 and Caspase-3 protein expression (Fig. 5A,E,G) in the LY group was significantly higher (*P* < 0.01). Compared with levels in the LY group, PCNA (Fig. 5A,C), cleaved Caspase-3 and Caspase-3 (Fig. 5A,E,G) protein expression levels in IEC-6 cells in the LDLY group did not differ significantly (*P* > 0.05). Caspase-3 and cleaved caspase-3 protein expression (Fig. 5A,E,G) in the HDLY group was significantly increased (*P* < 0.05). After the addition of 10 µmol/L MK-2206 2HCL, PCNA protein expression (Fig. 5B,D) in IEC 6 cells in the MK group was significantly lower than that in the CK group (*P* < 0.01); however, there was no difference in cleaved Caspase-3 and Caspase-3 protein expression (Fig. 5B,F,H) (*P* > 0.05). Compared with levels in the MK group, PCNA protein expression (Fig. 5B,D) did not differ significantly in the LDMK group (*P* > 0.05). However, Caspase-3 and cleaved Caspase-3 protein expression (Fig. 5B,F,H) was significantly higher in the LDMK group than in the MK group (*P* < 0.01). In the HDMK group, PCNA protein expression (Fig. 5B,D) was significantly decreased (*P* < 0.05), cleaved Caspase-3 and Caspase-3 protein expression (Fig. 5B,F,H) was significantly increased (*P* < 0.01).

After the specific inhibition of PI3K signaling, PCNA protein expression in IEC-6 cells decreased significantly and Caspase-3 protein expression increased significantly. Supplementation with 0.8 mmol/L boron did not affect PCNA and Caspase-3 protein expression levels in IEC-6 cells, while 40 mmol/L boron significantly increased Caspase-3 protein expression. After the specific inhibition of Akt signaling, PCNA protein expression in IEC-6 cells decreased significantly. Supplementation with 0.8 mmol/L boron did not increase PCNA protein expression in IEC-6 cells but significantly increased Caspase-3 protein expression. A dose of 40 mmol/L boron did not affect the protein expression levels of PCNA and Caspase-3. These results suggested that PI3K and Akt signaling mediated the effects of 0.8 mmol/L on PCNA and Caspase-3 protein levels in IEC-6 cells.

## Discussion

### The PI3K-Akt signaling pathway mediates the effects of boron on intestinal barrier function in rat intestinal epithelial cells

The small intestine is the main organ involved in animal digestion^17^. The intestinal absorption function depends on the structural integrity of the intestinal mucosa^18,19^. Secreted tight junction proteins are important components of the small intestinal mechanical barrier and immune function, and their expression levels can directly reflect small intestinal barrier function^20,21^. The tight junction is an important part of the lateral junction of small intestinal epithelial cells, with a decisive influence on intestinal mucosal permeability. It is mainly composed of several transmembrane proteins, cell solute proteins and their cytoskeletal components, such as Occludin, Claudins, and Zonula occludens (ZOs)^22^. Occludin and ZO-1 have obvious roles in maintaining tight junctions and intestinal mucosal permeability^23^. Changes in Occludin and ZO-1 expression could reflect the stability of tight junctions between intestinal epithelial cells and the permeability of the intestinal mucosa^24^.

Trace elements could affect the expression of Occludin, and ZO-1 in the intestinal mucosa, thereby influencing intestinal mucosal barrier function. For example, Xie et al.^25^ found that 20 µmol/L Zn can enhance the expression of the tight junction proteins ZO-1, Claudins-1, and Occludin at the protein and mRNA levels in duck primary intestinal epithelial cells. Zinc can ameliorate the intestinal barrier damage caused by *Shigella* by regulating the expression of Occludin^26^. Qiao et al.^27^ demonstrated that supplementation with 1 mg/kg selenium nanoparticles significantly increases the expression of Occludin and Claudin-1 in the jejunum in mice, thereby enhancing jejunal barrier function^28^. The expression levels of ZO-1, and Occludin in the duodenum of rats increased significantly by the addition of 40 and 80 mg/L boron to drinking water, and the intestinal barrier function was improved^14^. Our results demonstrated that 0.8 mmol/L boron promotes the secretion of and Occludin in IEC-6 cells, while 40 mmol/L boron inhibits the secretion of ZO-1 and Occludin, suggesting that low-dose boron can enhance the immune function and barrier function of small intestinal epithelial cells, while high doses of boron produce harmful effects. Our results confirm those of previous studies at the cellular level.

Tight junction proteins not only maintain the barrier between cells but also participate in the transmission of information between cells. They are associated with a variety of intracellular signaling molecules^29^. The transmission of information by tight junction proteins is controlled by a variety of signal transduction pathways, such as mitogen-activated protein kinase (MAPK) and PI3K/Akt, which affect the expression of tight junction proteins, control protein synthesis, and ultimately affect information transmission^30,31^. PI3K/Akt is an important intracellular signaling pathways and plays a key role in regulating the growth, proliferation, differentiation, and apoptosis of various cells^32,33^. PI3K is a dimer of regulatory subunit P85 and catalytic subunit P110, which binds to growth factor receptor to alter the protein structure and activate Akt, thereby activating or inhibiting a series of downstream factors involved in the regulation of cell proliferation, differentiation, apoptosis, and migration^34^.

PI3K/Akt could mediate the effects of various factors on the expression of Occludin and ZO-1. Dietary supplementation with 10 mg/kg baicalin can stimulate the PI3K/Akt signaling pathway in the intestinal tract of chicks and increase the expression levels of ZO-1 and Occludin in the intestinal tract, suggesting that the PI3K/AKT signaling pathway is involved in the beneficial effects of baicalin on the intestinal mucosal barrier of chicks^35^. Dietary supplementation with 0.75 g/kg glutamine can induce the up-regulation of PI3K/Akt signaling pathway-related protein expression, thereby enhancing intestinal barrier function^36^. The addition of 50 µmol/L resveratrol significantly increased the expression of intestinal tight junction proteins (Claudin-1, Occludin, and ZO-1); however, this effect of resveratrol was inhibited by blocking the PI3K/Akt pathway, suggesting that PI3K/Akt mediates the effect of resveratrol on intestinal permeability in pigs^37^. Our results demonstrated that when PI3K signaling was specifically blocked, 0.8 mmol/L boron did not promote the synthesis and secretion of Occludin and 40 mmol/L boron did not inhibit the expression of Occludin and ZO-1. Recent studies on the distribution characteristics of intestinal tight junction in animals have found that in normal cells^38,39^. Tight junction is distributed in continuous monolayer on the cell membrane. However, after intestinal injury, the distribution and shape of tight junction will show irregular zigzag shape and other features, and its connections will also show gaps, thus enhancing the permeability of cells, affecting intestinal absorption and barrier function. Therefore, we speculate that supplementation with different doses of boron may affect the barrier function of IEC-6 cells by affecting the content and distribution of tight junction proteins.

These results indicate that the PI3K/Akt signaling pathway mediates the effects of different doses of boron on the immune and barrier functions of small intestinal epithelial cells. It is possible that different doses of boron activate or inhibit the PI3K/Akt signaling pathway via solute carrier family 4 member 11 (*NaBC1*) (*NaBC1* is a boric acid transporter widely expressed in animal tissues. Studies have shown that *NaBC1* can affect cell proliferation and apoptosis via a PI3K-independent pathway^40^), thereby affecting the transcription, synthesis, and secretion of Occludin and ZO-1 in small intestinal epithelial cells.

### PI3K-Akt signaling mediates the effects of boron on proliferation and apoptosis in rat intestinal epithelial cells

Cell proliferation and apoptosis are important intracellular events that maintain normal growth and development and dynamically balance the development and repair of small intestine in rats. *PCNA* is a nuclear proliferation antigen and a helper protein of DNA polymerase, and its expression level reflects the state of cell proliferation^41^. Caspase-3 is a key enzyme in apoptosis; the level of caspase-3 reflects the status of apoptosis^42^. Many studies have shown that different doses of boron can affect the proliferation and apoptosis of various animal cells. Wang et al. ^10^ demonstrated that 0.4 mmol/L boron increases the proportion of CD4 + and CD8 + T lymphocytes, the contents of interleukin-2 and interferon-γ, and the protein expression of PCNA and decreases the rate of apoptosis in lymphocytes. However, supplementation with 40 mmol/L boron had the opposite effect, suggesting that low-dose boron could enhance lymphocyte immune function and promote proliferation, while high-dose boron inhibits lymphocyte proliferation and weakens immune function. Capati et al.^43^ have shown that 0.1 mmol/L boron promotes craniofacial development protein 1 and stromal cell-derived factor 4 in mammalian osteoblasts. mRNA expression levels of proliferative protein kinase 1, catenin-α1, and collagen-α1 accelerated the proliferation and differentiation of Osteoblastic cells. Zhang et al.^44^. reported that 0.01 mmol/L boron can increase the proliferation rate of ostrich splenic lymphocytes, reduce the rate of apoptosis and interferon-α expression levels, and inhibit the apoptosis of ostrich lymphocytes. Jin et al.^45^ further found that drinking water supplemented with 10 mg/L and 20 mg/L boron can promote the expression of *PCNA* and increase the levels of thymosin α1 and Glutathione peroxidase in thymus tissue, suggesting that adding an appropriate amount of boron can promote the proliferation of rat thymocytes and enhance the immune and antioxidant functions of the thymus. In our study, 0.8 mmol/L boron significantly increased the proportion of IEC-6 cells in the G2/M phase, cell proliferation rate, and *PCNA* mRNA and protein expression levels and decreased the proportions of cells in G0/G1 and S phase, apoptosis rate, cleaved caspase-3 levels and caspase-3 mRNA and protein expression levels. However, 40 mmol/L boron supplementation significantly decreased the proportions of IEC-6 cells in the G2/M phase, cell proliferation rate, and *PCNA* mRNA and protein expression levels and increased cleaved caspase-3 and caspase-3 protein expression, suggesting that low-dose boron supplementation can promote the proliferation of small intestinal epithelial cells in rats, while high-dose boron can promote the apoptosis of small intestinal epithelial cells in rats.

As an important intracellular signaling pathway, PI3K/Akt can mediate the effects of various factors on the proliferation and apoptosis of small intestinal epithelial cells^15^. He et al.^46^. showed that when the PI3K/Akt signaling pathway was blocked, downstream molecules could not be activated, resulting in an increase in the number of apoptotic cells. Costantini et al.^47^ found that Caco-2 human enterocytes were blocked in G0/G1 phase and the cell cycle was blocked after PI3K signaling was blocked. Bishnupuri et al.^48^ reported that when Akt was specifically blocked, the addition of canuridine did not affect the rates of apoptosis and proliferation in human colorectal cancer cells, suggesting that canuridine may enhance the activity of β-catenin via the PI3K/Akt signaling pathway, thereby affecting proliferation and apoptosis in human colorectal cancer cells. In addition, the subcutaneous injection of artesunate in rats can activate the PI3K/Akt signaling pathway, thereby promoting the expression of *Bax, LC3II/LC3I*, and Beclin-1 in rat chondrocytes, ultimately inhibiting chondrocyte proliferation and alleviating rheumatoid arthritis^49^. In our study, after specifically blocking PI3K signaling, the proliferation rate, *PCNA* mRNA and protein expression levels, and relative frequency of cells in the G2/M phase were significantly decreased, while caspase-3 mRNA and protein expression levels and cleaved caspase-3 levels were significantly increased. Supplementation with 0.8 mmol/L boron significantly decreased the proportion of G2/M cells, cell proliferation rate, and *PCNA* mRNA and protein expression levels but had no effects on the proportion of S-phase cells, rate of apoptosis, cleaved caspase-3 and caspase-3 mRNA and protein expression levels. The proportions of G0/G1 and G2/M cells, proliferation rate, apoptosis rate, and expression levels of *PCNA* and Caspase-3 were not affected by 40 mmol/L boron. After specifically blocking Akt signally, the proportion of G0/G1 phase cells and rate of apoptosis increased significantly, while the proportion of G2/M phase cells, proliferation rate, and *PCNA* mRNA and protein expression levels decreased significantly. Supplementation with 0.8 mmol/L boron did not reduce the proportion of G0/G1, S and G2/M phase cells and apoptosis rate. The expression of *PCNA* mRNA and protein was not decreased. The addition of 40 mmol/L boron did not decrease the G2/M phase cell percentage or cell proliferation rate and did not affect the apoptosis rate, cleaved caspase-3 and caspase-3 mRNA and protein expression levels. The current results indicate that the PI3K/Akt signaling pathway mediates the effect of low-dose boron on the proliferation and apoptosis of IEC-6 cells, while high-dose boron has a weak effect on the apoptosis of IEC-6 cells. Boron has estrogen-like effects and therefore it is possible that boron activates the PI3K/Akt signaling pathway via the estrogen receptor *ERβ*, thereby affecting the intracellular mitochondrial apoptosis signaling pathway and ultimately affecting cell proliferation and apoptosis^50^.

## Conclusion

After blocking PI3K or Akt signaling, 0.8 mmol/L boron did not affect levels of Occludin, *PCNA* mRNA and protein expression levels, the apoptosis rate, or caspase-3 mRNA and protein expression levels in IEC-6 cells. However, the proliferation rate and *PCNA* expression at the mRNA and protein levels decreased significantly. After blocking PI3K or Akt signaling, the addition of 40 mmol/L boron did not affect ZO-1 and Occludin levels, the proportions of G0/G1 and G2/M cells, or the cell proliferation rate and did not affect the apoptosis rate or the expression of *PCNA* and caspase-3. These results suggested that the PI3K/Akt signaling pathway mediates the effects of low-dose boron on immune function, barrier function, and cell proliferation and apoptosis in IEC-6 cells, while the regulatory effect of high-dose boron on the proliferation and apoptosis of IEC-6 cells is not obvious.

### Supplementary Information

Supplementary Information.

## Data Availability

The datasets generated during this study are available from the corresponding author on reasonable request.

## Abbreviations

PI3K: Phosphatidylinositol kinase
AKT: Protein kinase B
ZO-1: Zonula occludens-1
PCNA: Proliferating cell nuclear antigen
PI: Propidium iodide
GAPDH: Glyceraldehyde 3-phosphate dehydrogenase
ZOs: Zonula occludens
MAPK: Mitogen activated protein kinase
NaBC1: Solute carrier family 4 member 11
CK: The control group
LD: The low dose boron (0.8 mmol/L) group
HD: The high dose boron (40 mmol/L) group
LDLY: The low dose boron (0.8 mmol/L) with PI3K inhibitor (LY294002) group
HDLY: The high dose boron (40 mmol/L) with PI3K inhibitor (LY294002) group
LDMK: The low dose boron (0.8 mmol/L) with AKT inhibitor (MK-2206 2HCL) group
HDMK: The high dose boron (0.8 mmol/L) with AKT inhibitor (MK-2206 2HCL) group
