# Supplementary material for: PI3K/Akt signaling pathway mediates the effect of low-dose boron on barrier function, proliferation and apoptosis in rat intestinal epithelial cells
Source: Sci Rep. 2024 Jan 3;14:393. doi: 10.1038/s41598-023-50800-2 (PMC10764725; doi:10.1038/s41598-023-50800-2)

Supplementary Table S(1). Sequence of proliferation and apoptosis-related gene primers

Gene name	mRNA ID	Primer Sequence	Product length （bp）	Tm（℃）
PCNA	NM_022381.3	F:TAAGGGCTGAAGATAATGCTGAT<br>R:CCTGTTCTGGGATTCCTAGTT	126	60
Caspase-3	NM_012922.2	F:AGAGCTGGACTGCGGTAT<br>R:GCAAACTGGTGCTCAAGG	106	60
GAPDH	NM_017008.4	F:GGCAAGTTCAACGGCACAG<br>R:TAGTCGCCTTCCCCGCCTCT	232	60

Raw data of Western blot

Supplementary Figure S(1). β-Actin-Figure5A

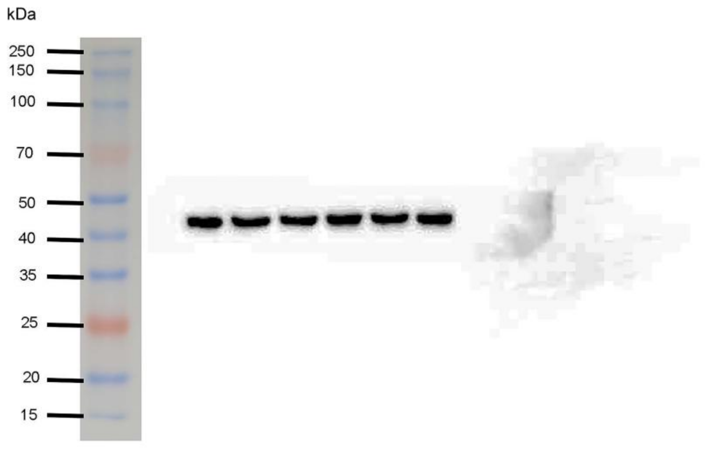

Supplementary Figure S(2). β-Actin-Figure5B

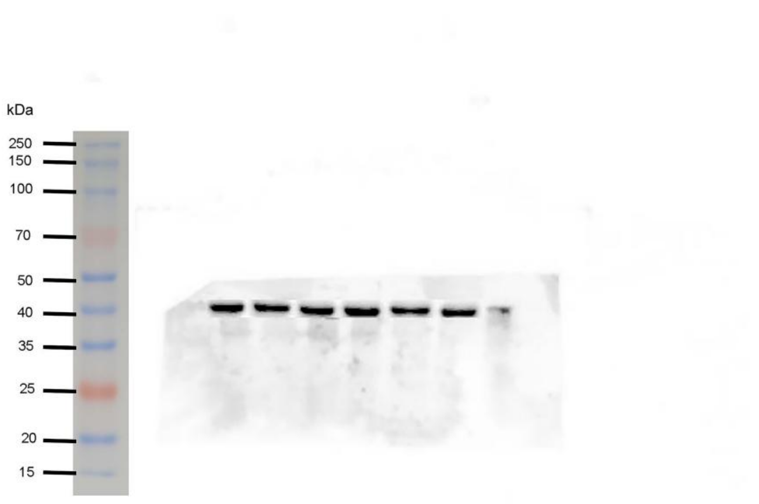

Supplementary Figure S(3). PCNA-Figure5A

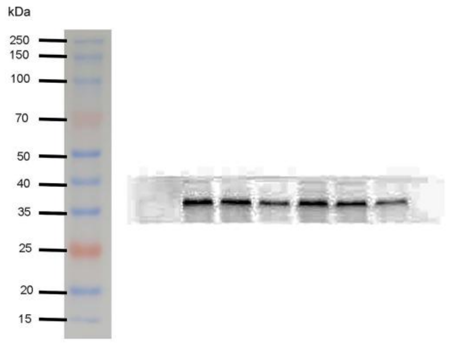

Supplementary Figure S(4). PCNA-Figure5A

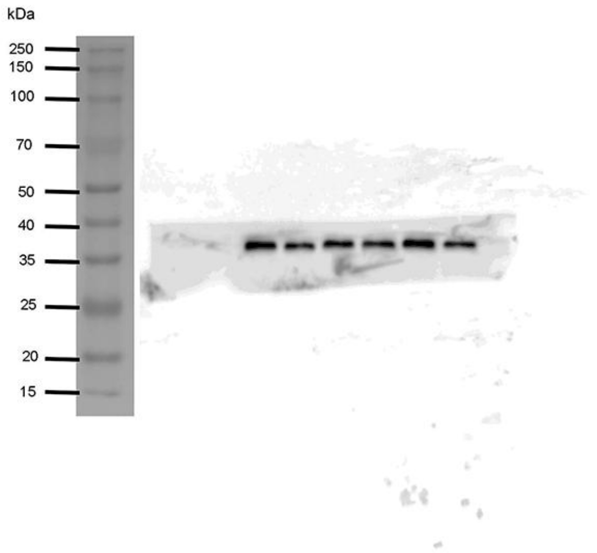

Supplementary Figure S(5). PCNA-Figure5B

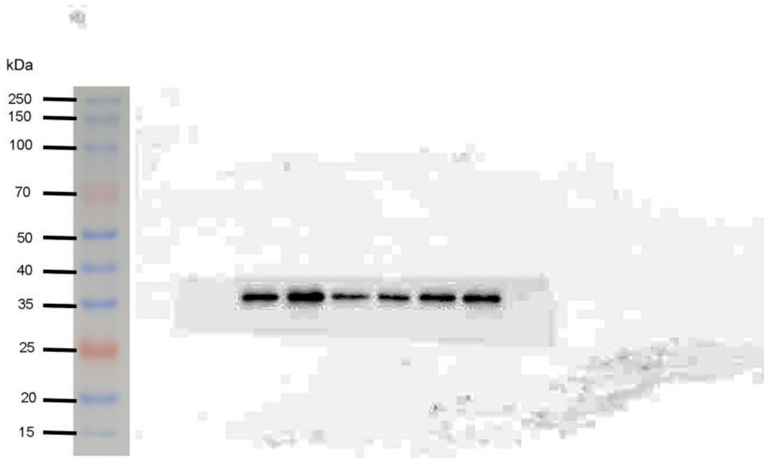

Supplementary Figure S(6). PCNA-Figure5B

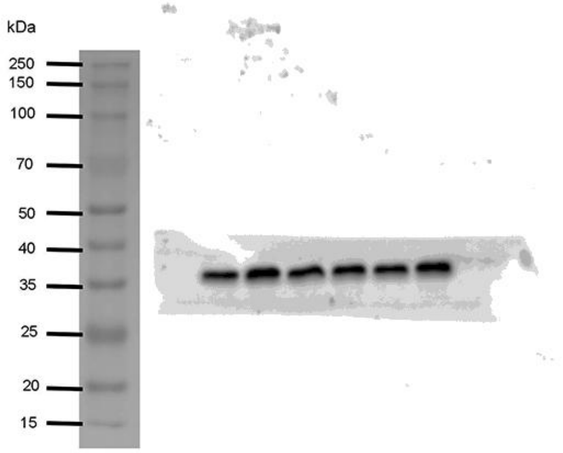

Supplementary Figure S(7). Caspase-3-Figure5A

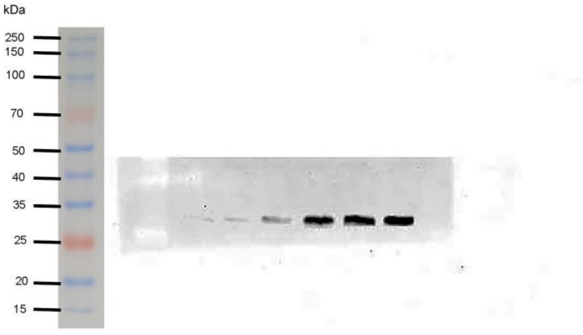

Supplementary Figure S(8). Caspase-3-Figure5A

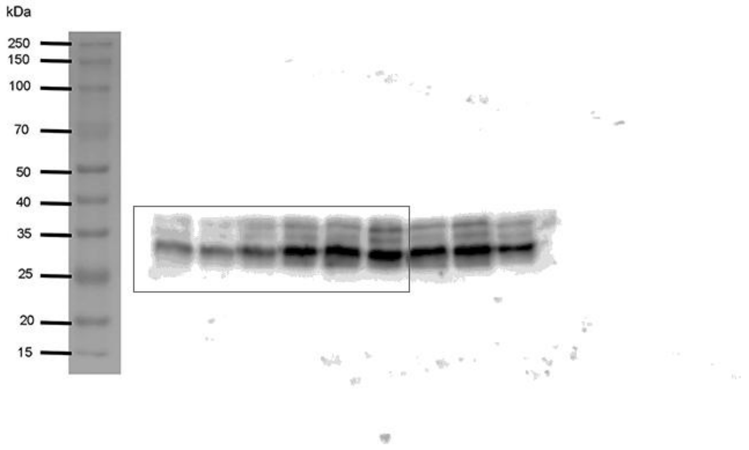

Supplementary Figure S(9). Caspase-3-Figure5B

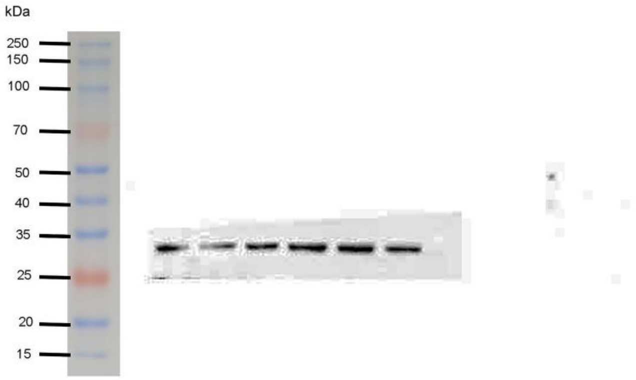

Supplementary Figure S(10). Caspase-3-Figure5B

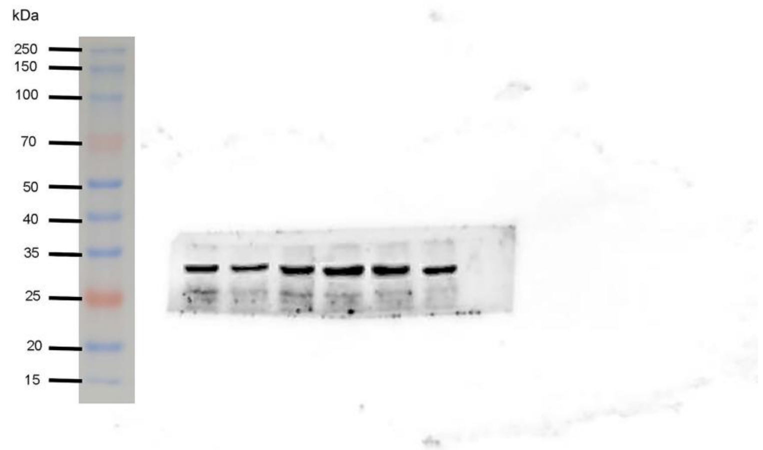

Supplementary Figure S(11). Cleaved Caspase-3-Figure5A

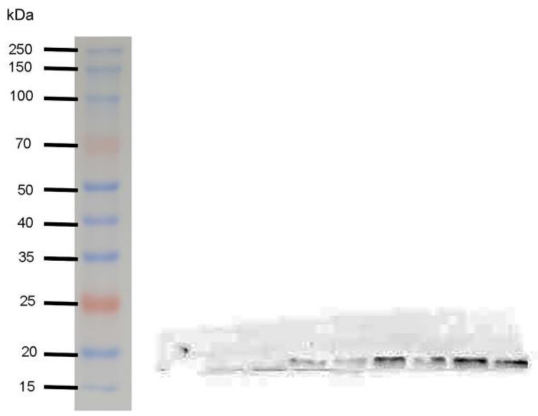

Supplementary Figure S(12). Cleaved Caspase-3-Figure5A

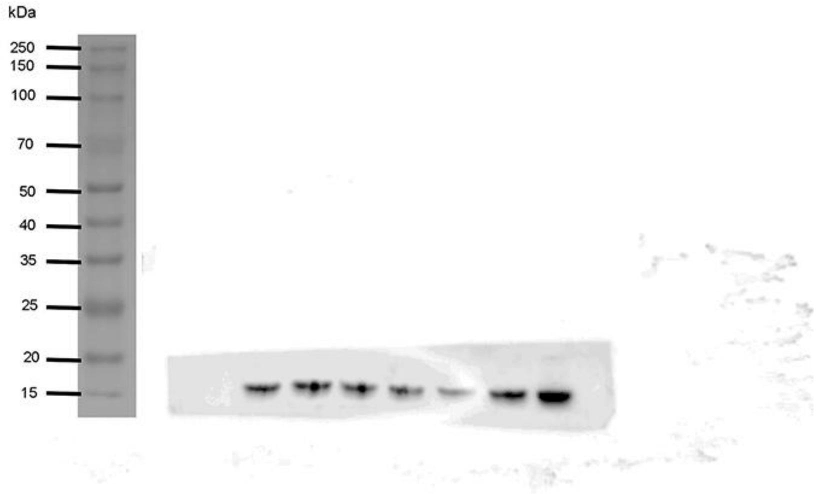

Supplementary Figure S(13). Cleaved Caspase-3-Figure5B

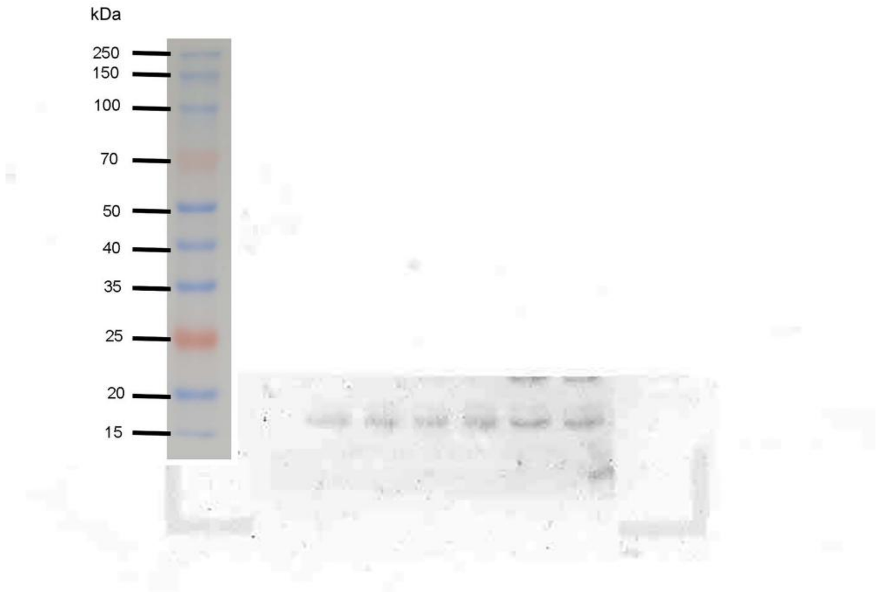

Supplementary Figure S(14). Cleaved Caspase-3-Figure5B

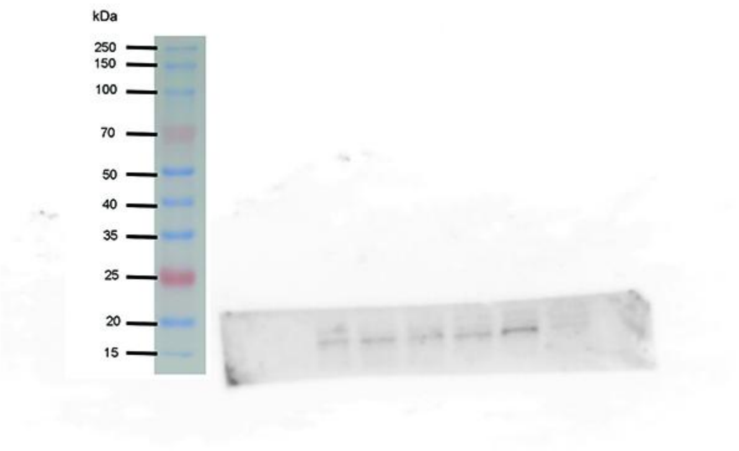

Recently experimental image

Supplementary Figure S(15). The PVDF film with the Marker before exposure

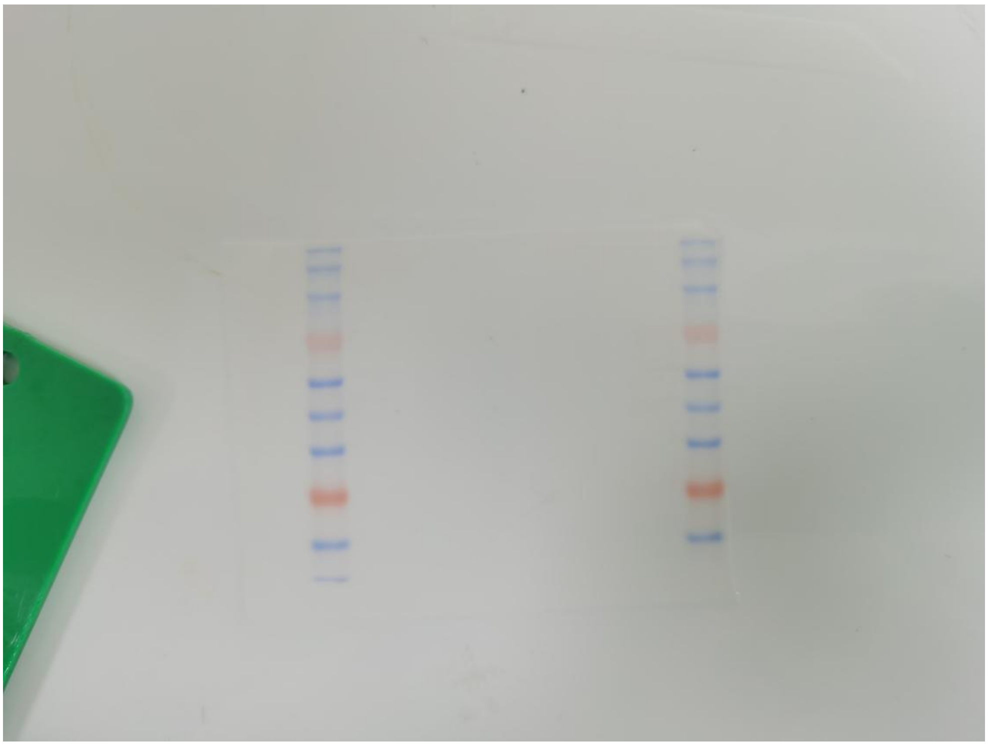

Supplementary Figure S(16). The image after exposure

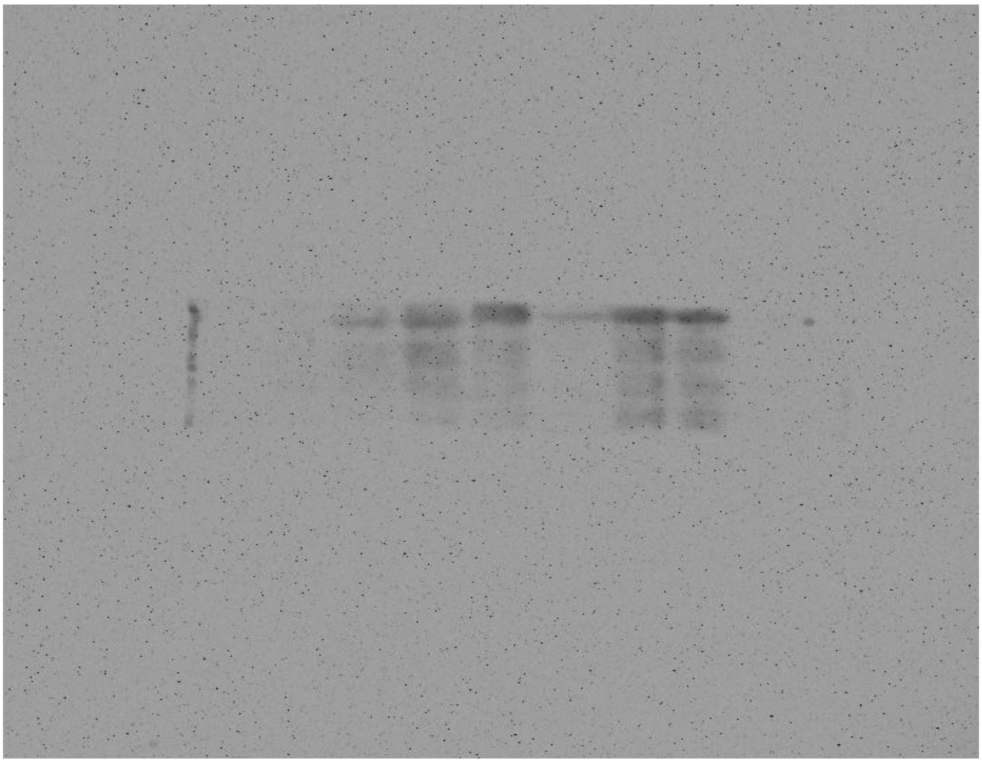

Supplement: Supplementary file 1 — Supplementary Information. [file 41598_2023_50800_MOESM1_ESM.pdf]
